# Leveraging Asymmetric Effects to Examine Dose and Long-term Effects of Helping Behaviors on Health Trajectories

**DOI:** 10.1093/geroni/igaf122.206

**Published:** 2025-12-31

**Authors:** Sae Hwang Han

**Affiliations:** The University of Texas at Austin, Austin, Texas, United States

## Abstract

Despite extensive research on how (pro)social engagement influences health, methodological approaches for examining dose changes and sustained long-term engagement in relation to health outcomes using observational data remain limited. This paper introduces a methodological approach for analyzing the health benefits of helping behaviors, emphasizing two key issues: (1) dynamic changes in helper role status and time commitment and (2) the long-term effects of sustained engagement. To this end, we extend the asymmetric fixed-effects modeling approach within a multilevel framework that allows for elucidating how intraindividual changes in helping behaviors—measured as both helper role status and time commitment—shape health trajectories. The proposed approach also incorporates strategies to address multiple sources of endogeneity and ensure proper temporal ordering of key variables, strengthening confidence in potential causal relationships. We demonstrate an empirical application of this approach in examining the relationship between helping behaviors and cognitive function using longitudinal data from the U.S. Health and Retirement Study (1998–2020; N = 31,303). The study findings reveal asymmetric effects, with cognitive benefits associated with role acquisition and increased time commitment, while role withdrawal and dose reduction were linked to cognitive decline. Notably, sustained engagement in moderate levels of helping (e.g., 2–4 weekly hours) yielded the greatest cognitive benefits, while abrupt changes, either in adopting or withdrawing from helping behaviors, were associated with less favorable outcomes. The proposed methodological approach provides a useful framework for evaluating modifiable lifestyle factors in gerontological research, offering new insights for designing interventions aimed at promoting health outcomes.

